# Model-free prognostication of non-linear time series

**DOI:** 10.1371/journal.pone.0341777

**Published:** 2026-02-02

**Authors:** Xiaoyong Wu, Shesh N. Rai, Georg F. Weber

**Affiliations:** 1 Biostatistics and Informatics Shared Resource, University of Cincinnati Cancer Center, College of Medicine, Cincinnati, Ohio, United States of America; 2 Department of Biostatistics, Cancer Data Science Center, University of Cincinnati College of Medicine, Health Informatics and Data Sciences, Cincinnati, Ohio, United States of America; 3 University of Cincinnati Cancer Center, College of Pharmacy, Cincinnati, Ohio, United States of America; University of Hamburg: Universitat Hamburg, GERMANY

## Abstract

**Objective:**

The COVID-19 pandemic has highlighted the importance of studying the course of infectious progression. Similar needs exist for time series of other origins. While models are commonly devised and fitted to the observed data, we recently demonstrated the feasibility to directly evaluate the noisy non-linear time series that characterize the occurrence. However, for practical utility, analytics alone has limited value. The requirement of forecasting – at least in the short term – needs to be met.

**Methods:**

We initially utilized normalized new infections per day (7-day moving average for cases per million inhabitants) from Our World in Data. We then validated our method in unrelated non-linear time series of stock markets and blowfly populations. We studied a novel model-independent time series approach, time lagged analyses, and feature-space plots incorporating the time-lagged data.

**Results:**

1) Machine learning on the basis of correlation coefficient, utilizing about 80% of the time series as training sets, was able to generate excellent predictions for progression. 2) Feature-space plots of normalized new cases versus autocorrelation and average mutual information required a form of dynamic calibration to correct for differences in scale among the axes. With that adjustment, the maximum local Lyapunov exponent displayed sharp spikes concomitantly with peaks of infectious spread. 3) The average mutual information over various time lags and wave lengths displayed divergence and sums of absolute values that were anticipatory to peaks in new infections.

**Conclusion:**

The study of non-linear time series with available techniques for observed complex data can extract characteristics that enable short-range forecasting without the need for model-building. Time-lagged analysis provides one suitable foundation. Among various approaches, machine learning achieved the best prognosticative results.

## Introduction

The spread of infectious diseases is determined by pathogen characteristics (contagion, virulence), host features (immunity, genetic variations, preexisting conditions), and community-directed countermeasures (social distancing, treatments, vaccines). From their exchanges, a complex pattern arises with spikes and troughs in new cases over time, resulting from the competition between factors that facilitate infectious progression and those that ameliorate it.

Diverse strategies have been applied to explain and understand this complexity of infectious propagation. The dissemination of COVID-19 has been modeled through networks of compartments [1,2], cellular automata [3,4], machine learning [5–7], artificial intelligence [8], and others. Commonly, those approaches build models on basic postulations, express them in sets of differential equations, and then map their results to the actual data. The subsequent match is as good as the initial (idealizing) premises and hypotheses permit.

The application of models, based on groups of assumptions and expressed in sets of differential equations, strives to enable prognostication, even over a somewhat extended range. In this time series forecasting, the choice of a model plays a crucial role in predicting future values based on previously observed values. Some of these assumptions are frequently applied. A standard model (S-I-R) divides the population into people who are susceptible to the disease (S), people who are infected by the disease and can spread it to others (I), and people who have recovered or died from the disease (R). S-I-R algorithms seek to detect changes in group size as people move from one group to another. They have been developed and modified in many ways [9,10]. Additional mathematical models use the basic reproduction number (R_0_), which represents the number of previously unexposed individuals who get infected by a new disease carrier. Those others then each go forth to spread it to more people at a similar frequency, and so on. However, all models have idealizations, and their predictions fail when disregarded (“minor”) contributing factors accrue to exert substantial influence.

Likewise, statistical forecasting approaches are fundamentally model-based methods and rely on the assumptions in a specific, structured statistical framework with multiple components (e.g., seasonality, trend) to capture underlying patterns in the time-series data. The autoregressive integrated moving average (ARIMA) models were applied to COVID-19 for predictions of epidemiological trends [11–13], prevalence and incidence [14], the spread of the virus and pandemic life cycle [15], and cumulative confirmed cases [16]. The ARIMA and Prophet models were used to predict a global omicron pandemic [17] and to forecast the number of confirmed and active cases [18]. TBATS (the ARIMA and trigonometric exponential smoothing state space model with Box–Cox transformation, ARMA errors, and trend and seasonal components) was employed to forecast disease severity in hospitalized patients [19], numbers of confirmed cases, deaths, and recoveries [20]. However, time progression series data, especially from epidemics, often do not fit into a specific statistical framework due to inherent complexities.

It is noteworthy that, in various events such as infectious progression, even shorter-term predictions may have a lot of value for the management of instabilities. Hence, their implementation is of practical benefit. Non-linear time progression series do not have closed-form solutions, and as such do not permit long-term predictions. In addition to modeling approaches, the analysis of the actually observed noisy and non-linear data is of critical importance [21]. This strategy does not require mechanistic premises or hypotheses at the outset. It seeks to extract patterns and characteristics from the numbers obtained through measurement in the field. We have recently shown the feasibility of acquiring meaningful information from such evaluations to aid decision making in the public health response [22]. Incompletely addressed, however, was the question to what extent predictions are possible within this framework.

We made the basic assumption that most time series, including contagion, do not represent a Markov process (in which the probability of each event depends only on the current one, and not on the prior history), but that the accumulated chronicle exerts a measurable influence on the future course. Further, the responses to any disturbances, which may arise, will be constrained by the attractor of the system. If this is the case, then – at the very least – short-term to mid-term predictions should be feasible from the study of observed non-linear occurrences. Similar considerations apply to other time series, such as stock market fluctuations and population dynamics.

Because time series data, if they are not entirely irregular, can be considered to represent waves, some tools from harmonic analysis are suitable for their description. Mutual information, in particular, provides a general redout for dependencies between variables and to be applicable to statistical questions [23]. Specifically for infectious spread, autocorrelation and average mutual information of lagged time series (wavelets) have previously been identified as very informative readouts for analytics [22]. When their values were plotted together with the rate of new cases in the same graph, local Lyapunov exponents served as descriptors for the relationships among these data sets. We therefore reasoned that correlation/autocorrelation and average mutual information of lagged time series, as well as Lyapunov exponents of their combined evaluation can provide a basis for forecasting without the reliance on prior model creation.

We also focus on utilizing correlation-based calculations, through machine learning and through empirical testing to develop a novel approach to prognostication that is not dependent on model building. The strategy does not assume any specific underlying statistical structure or generative model for the time series data. Our method leverages historical data to make predictions about the future data by identifying patterns and relationships within the collected data. In this regard, it separates itself from prior efforts [11–20].

## Methods

### Source data

We analyze the new infections per day, as the 7-day moving averages of the rates per million inhabitants. The source data utilized for the present analysis came from Our World in Data (https://ourworldindata.org/coronavirus) and were retrieved until either May 2022 or March 2023. This study utilizes deidentified data from the public domain. No consent was mandated for participation or publication. All methods were carried out in accordance with relevant guidelines and regulations. The experimental protocols did not require approval from an institutional and/or licensing committee.

### Univariate wavelet analysis

Wavelet assessment transforms the time series data from the time domain to the frequency domain. It can be applied to a single time series (univariate wavelet analysis). The calculations for wavelet analyses of new infections were done in R in an exploratory manner to assess the presence of periodic structure. In WaveletComp, the null hypothesis, that there is no periodicity in the series, is tested via p-values obtained from simulation, where the model to be simulated can be chosen from a range of options. The algorithm analyzes the frequency structure of uni- or bivariate time series using the Morlet wavelet [24]. As an important evaluation tool to study periodic phenomena in time series, wavelets are particularly useful in the presence of potential frequency changes across time. We conducted an assessment of lagged data (t versus t + 10 through t versus t + 90) for joint periodicity. The string lengths ranged from 100 to 400 days. Average mutual information and autocorrelation were calculated in R.

### Average mutual information

Mutual information uses probabilities (the information one data point gives about the other) to assess correlation. When there is a relationship between two variables (as is the case in lagged time series), one contains information about the other [25,26]. The average mutual information (AMI) represents a non-linear correlation function, which indicates how much common information is shared by the measurements at a particular time t and a later time t+τ in a time series $\left(x_{\text{1}},x_{\text{2}},\ldots ,x_{N}\right)$. The AMI for a given time delay τ is defined as


$$AMI(\tau )=\sum_{ij}p_{ij}\text{log}\left(\frac{p_{ij}}{p_{i}p_{j}}\right)$$

where $p_{i}$ is the probability that $x_{t}$ is in bin i, $p_{j}$ is the probability that $x_{t+\tau }$ is in bin j, and $p_{ij}$ is the joint probability that $x_{t}$ is in bin i and $x_{t+\tau }$ is in bin j. The sum of absolute AMI values was calculated as


$$\sum_{range}\left|AMI(t)\right|$$

with the range entailing either all covered lags for one wavelength or all covered wave lengths for one lag where *t* represents specific date. The AMI was calculated with the mutual function R package tseriesChaos [24].

### Autocorrelation

A time series $\left(x_{\text{1}},x_{\text{2}},\ldots ,x_{N}\right)$ sometimes has properties, due to which earlier values display some relation to later values. The autocorrelation statistic measures the degree of that affiliation as it refers to linear dependence. The magnitude of its dimensionless number reflects the extent of similarity. The formula for autocorrelation $R_{m}$ at lag m is the ratio of autocovariance and variance


$$R_{m}=\;\frac{\sum_{\text{t}=\text{1}}^{\text{N}-\text{m}}\left(x_{t}-\overset{―}{x})(x_{t+m}-\overset{―}{x}\right)\;}{\sum_{\text{t}=\text{1}}^{\text{N}}{\left(x_{t}-\overset{―}{x}\right)}^{\text{2}}\;}$$

where $\overset{―}{x}=\;\frac{\text{1}}{N}\sum_{i=\text{1}}^{N}x_{i}$.

### Correlation coefficient

Given data $\left(x_{\text{1}},\cdots ,x_{n}\right)$ and$\left(y_{\text{1}},\cdots ,y_{n}\right)$, the correlation coefficient can be calculated by


$$\rho \left(\left(x_{\text{1}},\cdots ,x_{n}\right),\;\left(y_{\text{1}},\cdots ,y_{n}\right)\right)=\;=\frac{\sum_{i=\text{1}}^{n}\left(x_{i}-\overset{―}{x}\right)\left(y_{i}-\overset{―}{y}\right)}{\sqrt{\left(\sum_{i=\text{1}}^{n}{\left(x_{i}-\overset{―}{x}\right)}^{\text{2}}\right)\left(\sum_{i=\text{1}}^{n}{\left(y_{i}-\overset{―}{y}\right)}^{\text{2}}\right)}},$$

where $\overset{―}{x}=\frac{\text{1}}{n}\sum_{i=\text{1}}^{n}x_{i}$ and $\overset{―}{y}=\frac{\text{1}}{n}\sum_{i=\text{1}}^{n}y_{i}$. Correlation coefficient ranges from −1 to +1, with +1 indicating perfect synchrony and −1 reflecting exact mirror images and with 0 indicating an absence of any correlation.

### Two-stage model-independent non-linear time series forecasting approach

Suppose that we observe the time series data $Z_{\text{1}},\cdots ,Z_{n-\text{1}}$ and aim to predict the future time series data $Z_{n}$, where $Z_{i}$ is the time series data on the $i^{th}$ day, i=1,⋯,n. Since the time series data had the scale of larger values, without loss of generality, let $Y_{i}=log\left(Z_{i}+\text{0}.\text{0}\text{5}\right),\;i=\text{1},\ldots ,n$, which compresses the scale of larger values, making the data more suitable for statistical analysis. Thus, the aim is equivalent to the prediction of the future time series data $Y_{n}$. For this, we propose a two-stage model-free approach for non-linear time series forecasting. To predict $Y_{n}$, it is enough to predict the difference between $Y_{n}$ and $Y_{n-\text{1}}$. Let $D_{i}=Y_{i}-Y_{i-\text{1}},i=\text{2},\ldots ,n$. A positive value indicates that the current value is greater than the previous value signifying an increase, a negative value indicates that the current value is less than the previous value signifying a decrease and a zero value indicates no change between the current and previous values.

At the first stage, we identify principal lags (PLs) by using linear models with only the first few lags as predictors to obtain less lags while keeping as much of causal effect of lags on data as possible. Specifically, for a given number q (assume q=19 here), we consider the following linear models with the p lags of $Y_{i-\text{1}}$ as predictors (p=2,…,q):


$$D_{i}=a_{\text{0}}+a_{\text{1}}Y_{i-\text{2}}+\cdots +a_{p}Y_{i-p-\text{1}}+\varepsilon_{i},\;i=p+\text{2},\ldots ,m+p+\text{1},$$

where mis the sample size (assumed m<n/4), $Y_{i-j}$ is the ${\left(j-\text{1}\right)}^{th}\;$ lag of $Y_{i-\text{1}},\;j=\text{2},\ldots ,p+\text{1}$, and $a_{j}$ is the unknown regression parameter, j=0,1,…,p, $\varepsilon_{i}$ follows a normal distribution with mean of 0 and a variance of $\sigma^{\text{2}}$.

The Bayesian Information Criterion (BIC) is a statistical measure used for model selection from a finite set of models. It is based on the likelihood function and incorporates a penalty term for the number of parameters in the model to avoid overfitting. BIC helps in identifying the model that best explains the data while balancing model complexity and goodness of fit. Lower BIC indicates a better model. We use the BIC as a measure for selecting lags. Let BIC(p)be the BIC in the linear model with the last p lags. If


$$\text{BIC}\left(p_{*}\right)=\underset{p=\text{2},\ldots ,q}{\text{min}}\text{BIC}(p),$$

then $Y_{i-\text{2}},\cdots ,Y_{i-p_{*}-\text{1}}$ are called the principal lags (PLs) of $Y_{i-\text{1}},\;i=p_{*}+\text{2},\ldots ,m+p+\text{1}$, and $p_{*}$ is called the number of PLs. Suppose that $p_{*}$ is the number of PLs of $Y_{i-\text{1}},\;i=p_{*}+\text{2},\ldots ,n-\text{1}$. Let $Y^{\left(i-\text{1}\right)}=\left(Y_{i-\text{2}},\cdots ,Y_{i-p_{*}-\text{1}}\right)$ denote the vector consisting of the PLs$i=p_{*}+\text{2},\ldots ,n-\text{1}$.

At the second stage, we propose the maximum correlation prediction for time series forecasting, which links the PLs at the current point to the PLs across all past time points by computing the correlation coefficients and find the optimal PLs that maximize the correlation coefficient. Specifically, for $i=p_{*}+\text{2},\cdots ,n-\text{1}$, the correlation coefficient of $Y^{\left(n-\text{1}\right)}$ and $Y^{\left(i-\text{1}\right)}$ given by


$$\rho \left(Y^{\left(n-\text{1}\right)},Y^{\left(i-\text{1}\right)}\right)=\frac{\sum_{j=\text{2}}^{p_{*}+\text{1}}\left(Y_{n-j}-{\overset{―}{Y}}_{n-\text{1}}\right)\left(Y_{i-j}-{\overset{―}{Y}}_{i-\text{1}}\right)}{\sqrt{\left(\sum_{k=\text{2}}^{p_{*}+\text{1}}{\left(Y_{n-j}-{\overset{―}{Y}}_{n-\text{1}}\right)}^{\text{2}}\right)\left(\sum_{k=\text{2}}^{p_{*}\text{1}}{\left(Y_{i-j}-{\overset{―}{Y}}_{i-\text{1}}\right)}^{\text{2}}\right)}},$$

where ${\overset{―}{Y}}_{n-\text{1}}=\frac{\text{1}}{p_{*}}\sum_{j=\text{2}}^{p_{*}+\text{1}}Y_{n-j}$ and ${\overset{―}{Y}}_{i-\text{1}}=\frac{\text{1}}{p_{*}}\sum_{j=\text{2}}^{p_{*}+\text{1}}Y_{i-j}$. If


$$\rho \left(Y^{\left(n-\text{1}\right)},Y^{\left(i*-\text{1}\right)}\right)=\underset{Y\in \left\{Y^{\left(i-\text{1}\right)}:i=p_{*}+\text{2}\ldots ,n-\text{1}\right\}}{\text{max}}\rho \left(Y^{\left(n-\text{1}\right)},Y\right),$$

then $Y^{\left(i*-\text{1}\right)}$ is called the most correlated vector of $Y^{\left(n-\text{1}\right)}$.

Note that the difference between $Y_{n}$ and $Y_{n-\text{1}}$ depends on its neighboring lags, i.e., PLs, while the difference between $Y_{i*}$ and $Y_{i*-\text{1}}$, depends on its neighboring PLs. Since $Y^{\left(n-\text{1}\right)}$ and $Y^{\left(i*-\text{1}\right)}$ are most correlated, it is reasonably assumed that $D_{n}$ will likely be close to $D_{i*}$., which implies that $D_{i*}$ can be used as the predicted value of $D_{n}$, i.e.,


$${\hat{D}}_{n}=D_{i*},$$

where ${\hat{D}}_{n}$ denotes the predicted difference between $Y_{n}$ and $Y_{n-\text{1}}$. Thus,


$${\hat{Y}}_{n}=Y_{n-\text{1}}+D_{i*},$$

where ${\hat{Y}}_{n}$ denotes the predicted value of $Y_{n}$. The algorithms for time series forecasting are presented in Supplement S1, Fig S1 in S1 File.

### Maximum correlation machine learning (MCML)

In time series analysis, a tie-based split is needed to separate the data into a training set and a test set. Here, the earlier portion of the data is used for training and the later portion is used for testing. This ensures that the prediction does not learn information from the future, when making predictions on the test set. Our machine learning process for forecasting involves the following steps:

Step 1. Split data into a training set denoted by $\Omega_{\text{0}}=\left(Y_{\text{1}},\cdots ,Y_{N}\right)$ and a testing set denoted by $\Omega_{\text{1}}=\left(Y_{N+\text{1}},\cdots ,Y_{N+M}\right)$, wherein $Y_{i}$ is the number of normalized new cases on the $i^{th}\;$day, i=1,⋯,N+M, and the training set includes 70–80% of the data while the test set includes the remaining 20–30% of the data.

Step 2. Apply the PLA to identify$Y_{i-\text{2}},\cdots ,Y_{i-p_{*}-\text{1}}$, the PLs of $Y_{i-\text{1}},\;i=p_{*}+\text{2},\ldots ,N$, and let $Y^{\left(i-\text{1}\right)}=\left(Y_{i-\text{2}},\cdots ,Y_{i-p_{*}-\text{1}}\right)$ denote the vector consisting of the PLs.

Step 3. Apply the maximum correlation prediction to calculate the most correlated vector of $Y^{\left(N\right)}=\left(Y_{N-\text{1}},\cdots ,Y_{N-p_{*}}\right)$


$$Y^{\left(i_{\text{1}}^{*}*-\text{1}\right)}=\underset{Y\in \left\{Y^{\left(i-\text{1}\right)}:i=p_{*}+\text{2},\ldots ,N\right\}}{\text{arg max}}\rho \left(Y^{\left(N\right)},Y\right).$$

and then calculate the predicted value of $Y_{N+\text{1}}$ through the equation


$${\hat{Y}}_{N+\text{1}}=Y_{N}+D_{i_{\text{1}}^{*}}.$$

Step 4. Similarly, calculate the next predicted values of $Y_{N+k}$ through the equation


$${\hat{Y}}_{N+k}={\hat{Y}}_{N+k-\text{1}}+D_{i_{k}^{*}},\;k=\text{2},\ldots ,\;M,$$

where $Y^{\left(i_{k}^{*}*-\text{1}\right)}$ is the most correlated vector of $Y^{\left(N+k-\text{2}\right)}≜\left({\hat{Y}}_{N+k-\text{3}},\ldots ,{\hat{Y}}_{N+k-p_{*}-\text{2}}\right)$.

Step 5. Calculate the three metrics: mean absolute error (MAE), root mean square error (RMSE) and mean absolute percentage error (MAPE) for evaluation of the accuracy, which are defined as follows:


$$MAE=\frac{\text{1}}{M}\sum_{i=N+\text{1}}^{N+M}\left|Y_{i}-{\hat{Y}}_{i}\right|,$$

$$RMSE=\sqrt{\frac{\text{1}}{n}\sum_{i=N+\text{1}}^{N+M}{\left(Y_{i}-{\hat{Y}}_{i}\right)}^{\text{2}}},$$

and


$$MAPE=\frac{\text{1}}{n}\sum_{i=N+\text{1}}^{N+M}\left|\frac{Y_{i}-{\hat{Y}}_{i}}{Y_{i}}\right|.$$

We compared the new method to conventional prediction algorithms. Autoregressive Integrated Moving Average (ARIMA) is a statistical model that combines autoregression (AR), differencing (I), and moving average (MA) to model and forecast time series data, especially when the data show trends or non-stationarity. ARMA Errors refers to modeling the residuals (errors) of a time series model using an ARMA (Autoregressive Moving Average) process to account for autocorrelation in the errors. Trigonometric Seasonality models seasonal patterns using sine and cosine functions to capture periodic fluctuations in time series data. Box-Cox Transformation represents a power transformation technique used to stabilize variance and make a time series more normally distributed, improving the performance of linear models. Trend and Seasonal Components (TBATS) is a time series forecasting model that includes Trigonometric seasonality, Box-Cox transformation, ARMA errors, Trend, and Seasonal components, suitable for complex seasonal patterns and multiple seasonalities. Threshold Autoregressive (TAR) is a nonlinear time series model where the data follow different autoregressive processes depending on whether the series crosses certain threshold values. Prophet Forecasting Model (PROPHET) constitutes a forecasting tool developed by Facebook that decomposes time series into trend, seasonality, and holiday effects, designed to handle missing data and outliers, and to be intuitive for analysts.

### Feature-space plots

Feature-space plots map 2 or more features of the time series. From the normalized numbers of new infections, their autocorrelation, and their average mutual information, we initially generated feature-space plots entailing various lags and chain lengths for the latter two readouts. In the case of the data sets under study here, we settled on sliding window durations of 150 days and time lags of 15 days.

For a discrete mapping x(t+1→F(x(t)), we calculated the local expansion of the flow by considering the difference between 2 trajectories. The local Lyapunov characteristic exponent λ can be approximated as


$$\lambda \;\approx \text{ln}(\left|x_{n+\text{1}}-y_{n+\text{1}}\right|/\left|x_{n}-y_{n}\right|)$$

for 2 points *x*_*n*_,*y*_*n*_ close to each other on the trajectory. In a multidimensional phase space, the maximum Lyapunov exponent (MLE) is the largest value among the pairwise comparisons. Notably, in the particular pairwise comparison of normalized new infections/autocorrelation, autocorrelation/average mutual information, and average mutual information/normalized new infections, a problem of scaling arose, because the mostly larger numbers along the ‘new infections’ axis flattened the trajectories. We applied a dynamic scaling approach, where autocorrelation (AC) and average mutual information (AMI) were brought into comparable ranges by multiplication with the half-maximal number of new cases during the window length (for autocorrelation+1 and directly for AMI).


$${AC}_{scaled}=(AC+\text{1})*(\frac{\text{1}}{\text{2}}\text{ max}({nc}_{wl}))$$

$${AMI}_{scaled}=AMI\;*(\frac{\text{1}}{\text{2}}\text{ max}({nc}_{wl}))$$

with *max()* = maximum of, *nc*_*wl*_ = normalized new cases (daily 7-day moving averages per million inhabitants) over the sliding window duration. Further scaling adjustments were explored as described under Results.

## Results

### Machine learning based on correlation analysis achieves model-free prediction

Based on our observation (S2 in S1 File) that data from time-lagged analysis have potential for anticipating time course progressions, we sought to strengthen the calculations by incorporating machine learning. Starting from observed time series data, the first four lagged variables were identified as principal variables for prediction of the difference between future new cases and past-to-current new cases. We divided the time course series into training (~80%) and test (~20%) sets, then applied the approach dubbed maximum correlation machine learning (MCML) to anticipate the new cases in the test sets. Specifically, we applied the time series values of a chosen sample size (n = 60) from the Australia dataset to the algorithms with the numbers of lags ranging from 2 to 19 and then identified the first 4 lagged variables as principal variables for prediction of the difference between future new cases and past-to-current new cases, because BIC was minimized when the number of lags was 4. The first 4 lagged variables were also identified as principal variables for prediction when we applied the time series values of sample sizes (n = 120, 200) to the linear models (S3, Fig S8 in S1 File). MCML predicted some time series patterns, such as peaks, with a shorter than 4-step time lag or advance (e.g., 2 days lag for peak in the Brazil dataset, 3 days advance for trough in the South Africa dataset, 1 day lag for peak in the Bangladesh dataset). Our MCML for non-linear time series forecasting might be further strengthened by using other hyperparameter tuning techniques or using machine learning based on additional metrics (e.g., kernels), which could guarantee future success.

**Fig 1:**
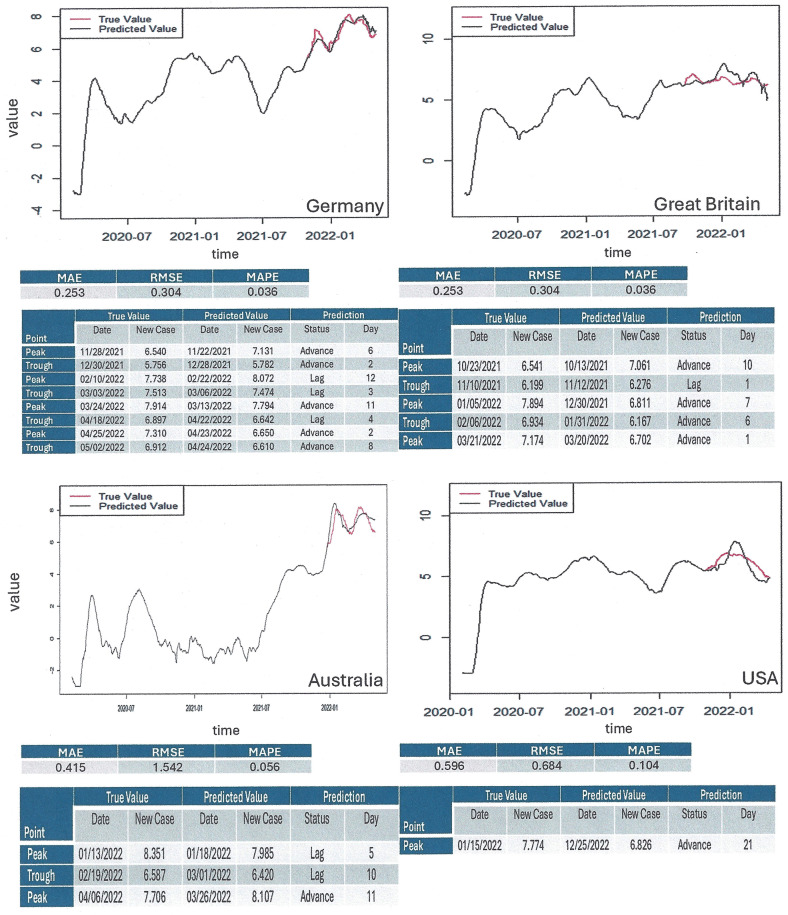
Machine learning for model-free prognostication. The time series of normalized new infections in 4 countries were utilized in a machine learning algorithm based on autocorrelation. The black and red color lines represent the true and anticipated numbers of new cases. All values are on the scale of log plus 0.05. The sizes of the training set and the testing set were chosen to be about 80% and 20%. The tables underneath the graphs display three distinct error calculations. MAE = mean absolute error, RMSE = root mean square error, MAPE = mean absolute percentage error. **Top left)** Prediction of peaks for the data Germany (n = 819) based on the training set (n = 634) and the testing set (n = 185). **Top right)** prediction of peaks for the data Great Britain (n = 814) based on the training set (n = 592) and the testing set (n = 222). **Bottom left)** prediction of peaks for the data Australia (n = 819) based on the training set (n = 689) and the testing set (n = 130). **Bottom right)** Prediction of peaks for the data USA (n = 802) based on the training set (n = 635) and the testing set (n = 167).

**Fig 2:**
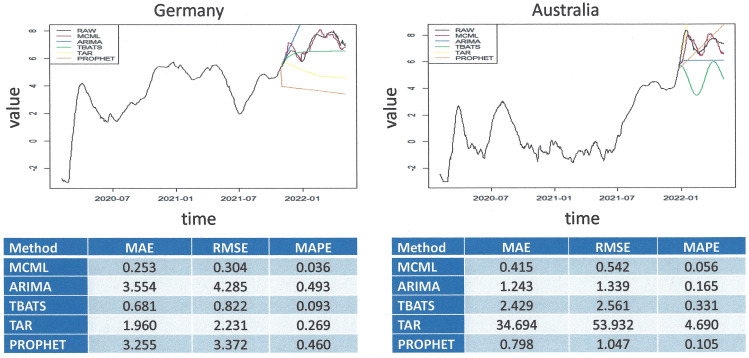
Validation of the machine learning for model-free prognostication. The figure provides the comparative performance results for the 5 methods. The MCML method had the best performance of all forecasting methods and generated forecasting results with the lowest MAE, MAPE, and RMSE in each case. **Top left)** Comparisons of time-series forecasting using our method (maximum correlation machine learning (MCML) with the autoregressive integrated moving average (ARIMA), trigonometric seasonality, Box-Cox transformation, ARMA errors, trend, and seasonal components (TBATS), threshold autoregressive (TAR), Prophet forecasting model (PROPHET) in the Germany dataset (n = 819) based on the training set (n = 634) and the testing set (n = 185). **Bottom left)** Comparisons of our method (MCML) to the conventional time series methods for the Germany dataset. **Top Right)** Comparisons of time-series forecasting using our method (MCML) with the autoregressive integrated moving average (ARIMA), trigonometric seasonality, Box-Cox transformation, ARMA errors, trend, and seasonal components (TBATS), threshold autoregressive (TAR), Prophet forecasting model (PROPHET) in the Australia dataset (n = 819) based on the training set (n = 689) and the testing set (n = 130). **Bottom right)** Comparisons of our method (MCML) to canonical time series methods for the Australia dataset.

Fig 1 (Fig 1) illustrates that this algorithm has the capacity to capture time series information from various source data with differing progressions, including trend and auto-correlation patterns. Good forecasting performance was achieved overall, with the best results (among 10 countries analyzed) having been obtained for predicting COVID-19 new cases from Australia and Germany. We also compared the new method to conventional prediction algorithms in terms of MAE, RMSE and MAPE. Among ARIMA, ARMA, TBATS, TAR, and PROPHET, none of the prior algorithms performed satisfactorily, and all fell short in comparison to MCML in terms of MAE, RMSE and MAPE (Fig 2).

**Fig 3:**
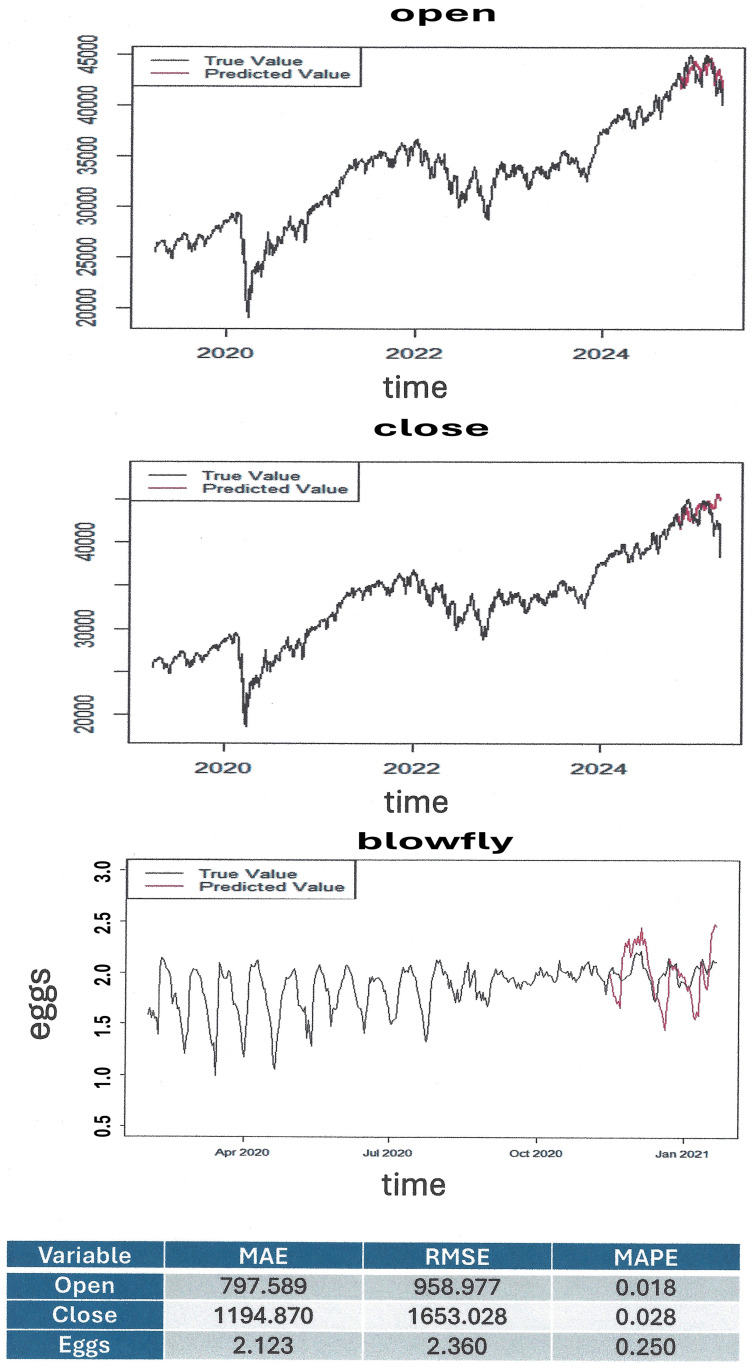
Analyses of time series from various sources. Non-linear time series data were obtained from financial markets and from entomology observations. **Top and middle)** Stock market fluctuations represent classical non-linear time series. We retrieved daily data for “INDEX_US_DOW JONES GLOBAL_DJIA” (n = 1521) and applied machine learning based on the training set (n = 1400) and the testing set (n = 121), where the black and red color line represent the true and predicted values of new cases, respectively. The y-axis values are unitless. **Top)** Prediction of peaks for “Open” **Middle)** Prediction of peaks for “Close”. **Bottom)** The Nicholson blowfly experiments were conducted in the 1950s with the intent of learning more about a sheep pest, the blowfly [31,32]. The data involve a system that is nonlinear, has time lags and might be described as non-stationary. Prediction for “eggs” in the data “blowfly97I” (n = 361) using machine learning based on the training set (n = 289) and the testing set (n = 72). **Table)** The table underneath the graphs display three distinct error calculations. MAE = mean absolute error, RMSE = root mean square error, MAPE = mean absolute percentage error.

The machine learning algorithm is generally applicable to non-linear time series, regardless of the data origin. Therefore, we validated it in financial fluctuations of the Dow-Jones Index as well as population dynamics of blowfly reproduction, both with convincing results (Fig 3). The MCML algorithm works well for time series forecasting when the series exhibits repeating patterns or local dynamics, where short-term behavior mirrors past similar periods. However, it experiences confines when limited data are available. Also, as is the case for all forecasting algorithms, MCML quality declines for very noisy data, which obscure the underlying patterns and relationships to be learned.

### Maximum local Lyapunov exponents from normalized feature-space plots spike with infection peaks

**Fig 4:**
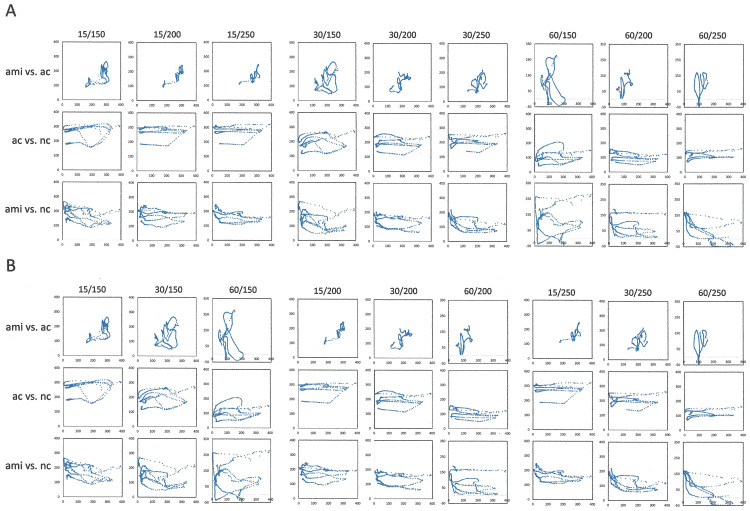
Feature-space plots for various sliding window durations and time lags. For the South Africa data, the three readouts, ac, ami, normalized new cases (nc) are graphed pairwise against each other. **A) increasing sliding window durations over 3 time lags. B) increasing time lags over 3 sliding window durations.**

**Fig 5:**
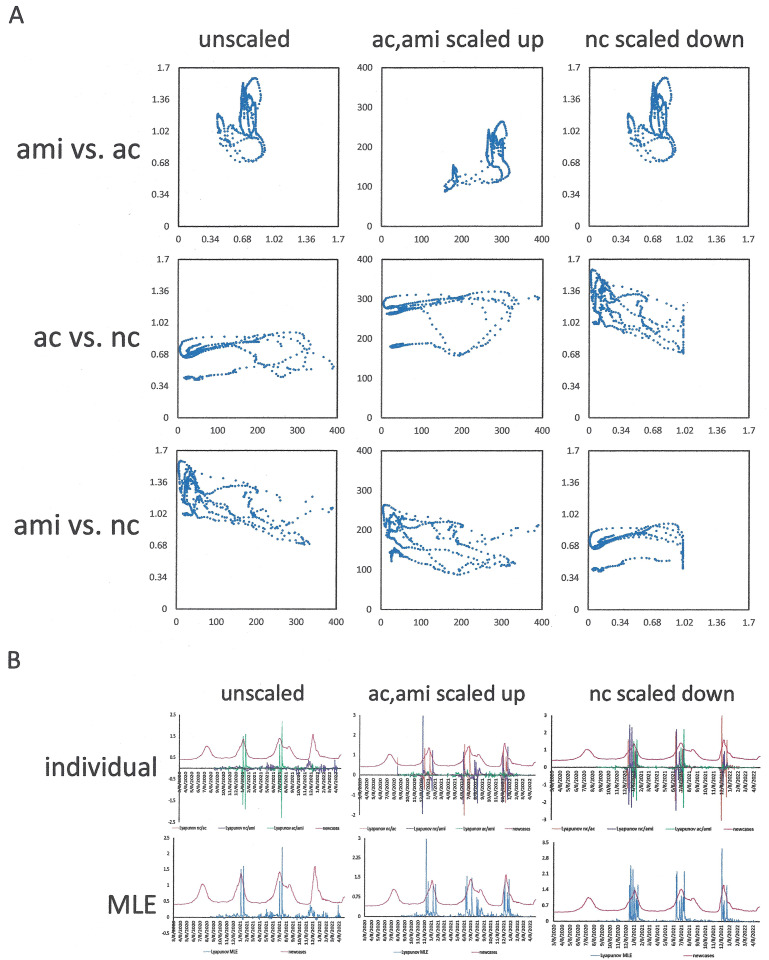
Feature-space plots and local Lyapunov exponents. **A,B)** The data for South Africa were analyzed. The panels on the left display the feature-space plots for the raw data. In the middle panels, ac + 1 and ami were scaled up by multiplication with the half-maximal value of new cases during the sliding window durations. In the right panels, the values for the new cases were divided by their maximum during the observation window. **A) Feature-space plots.** Shown are the pairwise feature-space plots for average mutual information (ami), autocorrelation (ac), and new cases (7-day moving average per million inhabitants) (nc), comprising string lengths of 150 days and time lags of 15 days. **B) Lyapunov exponents over time.** In the upper panel, the individual Lyapunov characteristic exponents are shown in comparison to the suitably scaled new cases over time, for each pair of readouts (i.e., pairwise among nc = new cases, ac = autocorrelation, ami = average mutual information). The red trace indicates the normalized new cases per day. The bottom panel displays the maximum Lyapunov exponents (MLE) over time (blue line) in comparison to the normalized new cases per day (red line).

**Fig 6:**
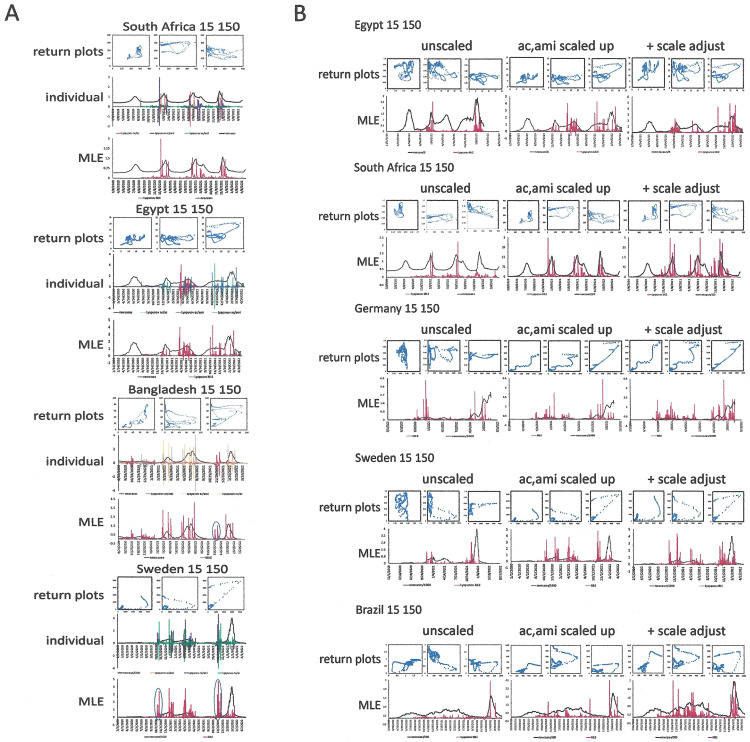
Return plot scaling and local Lyapunov exponents. **A) Lyapunov exponents over time in several countries.** For the analysis, ac + 1 and ami were scaled up by multiplication with the half-maximal value of new cases during the sliding window durations. The string lengths are 150 days and time lags are 15 days. MLE = maximum Lyapunov exponent. **B) Scaling of feature-space plots and maximum Lyapunov exponents.** All univariate wavelet analyses are based on sliding window durations of 150 days and time lags of 15 days. Each row represents a country. The left column displays unscaled data, the middle column shows the scaling up of ac and ami (as in A)), the right column displays the added step of an arbitrary scale adjustment. This step entails (consecutive to the described prior steps) in Egypt ami*2, in South Africa nc*0.75, in Germany ami*1.7, in Sweden ami*2, in Brazil ac*2.2.

In time-lagged analysis, important conclusions can be inferred from the assessments of autocorrelation and average mutual information. For the purpose of prediction, the results from both algorithms are relevant in correlation to the peaks of the normalized new cases (NC). Feature-space plots with each of these three readouts on the axes (AC, AMI, NC) may be of importance. In this depiction, a rapid increase or decrease in new infections is reflected in a close-to straight line, oscillations generate a near-toroid attractor, while successful management shrinks the torus and moves it closer to the origin. However, sliding window durations and time lags need to be chosen suitably (Fig 4). Also, a problem of scaling arises. The values for autocorrelation are strictly confined to the interval −1 to +1, and the average mutual information tends to be in the low single digits (while not applied here, it can be normalized to an interval of [0,1] when expressed as the mutual information of the variables x and y, divided by the mean entropy of x and y). By contrast, the new cases, even consecutive to having been scaled in the form of 7-day moving averages per million inhabitants, can be in the hundreds. Resultantly, the calculation of maximum local Lyapunov exponents (MLE) is skewed in one dimension of the 3-dimensional return plot. Therefore, another scaling step is required for graphing the readouts for these three parameters in one return plot. The simple normalization of the new cases-axis by division through the highest number over the entire observation period may be meaningful retrospectively, but this adjustment does not support the progressive day-by-day analysis, in which another, higher peak may arise with every new data point. We therefore sought to apply a normalization that covers only the observation period used for the time-lagged analysis (i.e., the observation window in days; as the length stays constant and the wavelet moves with every new data point, this equates to a dynamic scaling that adjusts day-by-day). Notably, dividing the normalized new cases by their largest value within the sliding window duration seemed to compress the high end of the spectrum near the maximum of 1.0 relative units (because, during an upswing, every day introduces a new highest value). By contrast, the scaling up of average mutual information and (autocorrelation + 1) by multiplication with the half-maximal number of new cases during the observation window brought the quantities for all three parameters onto comparable scales, and in consequence the spikes of the MLE values correlated much better with the peaks of new cases per day than did the MLE for the unscaled graphs (Fig 5A, 5B). When this scaling method was corroborated on multiple data sets, the MLE values spiked coincidingly with or ahead of peaks in new infections (Fig 6A). Even after these adjustments, further scrutiny identified minor remaining discrepancies in the scales of the return plot axes. We therefore tested an additional arbitrary scaling step, which used multiplication with a constant to bring to scale the values of one suboptimally matched axis. This achieved further improvement in aligning the MLE spikes with the peaks in normalized new cases (Fig 6D).

We approximated a first derivative (change between consecutive days) by calculating the value of t + 1 minus the value of t for the normalized new cases, the autocorrelation, and the average mutual information (S4, Fig S9A in S1 File). While the daily changes in autocorrelation were fairly reflective of the daily changes in new cases, the daily changes in average mutual information contained too much noise to be useful. The differentials in local Lyapunov exponents for each axis of the 3-dimensional return plot impressed as uninformative (S4, Fig S9B in S1 File).

## Discussion

The analysis of complex time series has increasingly relied on model building, which makes assumptions on underlying connections, composes non-linear differential equations, runs them through computer analysis, and matches them to the original data. Close congruence is taken as confirmation that the model has validity. Deviations are seen as an impetus to add variables and equations to the model. This approach starts with assumptions and ends with observed data. While it has been widely practiced, its results are contingent with the accuracy of the model built. There is feasibility to obtain information directly from the observed data without relying on model building first.

For the analysis of non-linear time series, lagged variables have proven useful for characterizing the curves of events versus time. Dynamic models (e.g., ARMA models, ARDL models, etc.) have been developed using linear combinations of various types of lagged variables. In the present investigation, we find that the values for average mutual information and autocorrelation/correlation (calculated from distinct lags of univariate wavelet analysis) can be processed to anticipate spikes in new infections. Rather than relying on a preconceived model, this strategy solely assumes that a) the time series under study does not represent a Markov chain but that its history influences and constrains its future development b) the response to disturbances is constrained by the attractor of the system. Hence, information gained from the past enables at least probabilistic projections on the future. Beyond infectious progression, this approach is applicable to time series of any origin (and has been validated here with data from financial markets and population dynamics).

Studies of complex occurrences have utilized harmonic analysis, adaptive landscapes, and conceptual spaces [27]. While the present study relies mostly on the first modality, it has incorporated conceptual spaces. Feature-space plots are adjustable to display maximum Lyapunov exponents that spike concomitantly with, or preceding to a peak in new infections. Proof-of-principle is established that forecasting of observed complex data is feasible on the basis of analytics, without the need for fitting models to the raw data first. There is opportunity for expansions of this approach, such as the inclusion of embedding dimensions or of Fourier analysis. Regarding the machine learning methodology, the inclusion of deep learning techniques can be explored [28–30].

**Fig 7:**
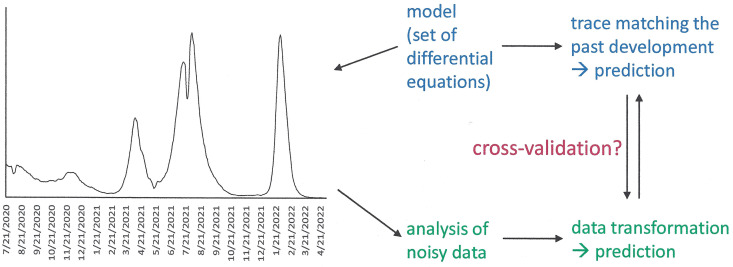
Analysis of complex time series. Conceptualization of alternative approaches to the study of non-linear data (the graph represents a time series measured in any applicable units). The top row (blue text) represents the model building approach, which makes assumptions, expresses them in sets of differential equations, and then tests their fit to existing measured data. The bottom row (green text) depicts the analysis of noisy complex data, which does not depend on model building but transforms the data for gaining insight. Strategies for cross-validation could improve the ability to make predictions (red text).

Enabling accurate prediction of an upcoming peak in disease dissemination requires investigations, as to whether the most suitable basis is provided by the differential equations of a model or by the analysis of observed non-linear data or by any combination that connects those distinct approaches. It is reasonable to assume that combinations of model-based analytics with direct analytics of the noisy non-linear data will have the best prospects for mapping and predicting the course of complex phenomena (Fig 7). The approaches outlined here are not limited to epidemics or pandemics but may also be of value for the assessment or the prediction of instabilities in a plethora of other non-linear time series.

## Conclusion

Non-linear time series do not have closed form solutions. Under existing paradigms, forecasting is achieved via fitting of models. Analytics of noisy, non-linear data can accomplish description, which implies the possibility of some prognostication. Machine learning on the basis of correlation coefficient, utilizing about 80% of the time series as training sets, was able to generate excellent predictions for the time series progression. Time-lagged analysis and feature-space plots require some adjustments of conventional approaches. They are partially successful.

## Supporting information

S1. File Supplement.
(DOCX)
