# Supplementary material for: Model-free prognostication of non-linear time series
Source: PLoS One. 2026 Feb 2;21(2):e0341777. doi: 10.1371/journal.pone.0341777 (PMC12863698; doi:10.1371/journal.pone.0341777)
Supplement: S1. File — Supplement. (DOCX) [file pone.0341777.s001.docx]

# Model-free prognostication of non-linear time series

## Supporting Information

Xiaoyong Wu, PhD^1,2^; Shesh N. Rai, PhD^1,2^; Georg F. Weber MD,PhD^3*^

^1^ Biostatistics and Informatics Shared Resource, University of Cincinnati Cancer Center, College of Medicine, Cincinnati, OH, USA

^2^ Cancer Data Science Center, University of Cincinnati College of Medicine, Department of Biostatistics, Health Informatics and Data Sciences, OH, USA

^3^ University of Cincinnati Cancer Center, College of Pharmacy, Cincinnati, OH, USA

# Supplement 1

**Figure S1. Time series forecasting algorithm. Top Panel)** Flow chart for the analysis. **Bottom Panel)** Correlation coefficients (CC) and differences (n=10). Suppose that we have observed the data for 10 days. These values are 1.871, 2.073, 1.917, 1.930, 2.013, 1.967, 1.930, 2.001, 2.059 and 1.823 on the date order. To predict $Y_{11}$, we calculate the differences between $Y_{i}$ and $Y_{i-1} (i=2,\ldots,10)$ and the correlation coefficients of $Y^{\left( 10 \right)}$ and all previous vectors $Y^{\left( i \right)} (i=5,\ldots,9)$. It is easily seen from the table that $\rho\left( Y^{\left( 10 \right)},Y^{\left( 7 \right)} \right)=0.543$, which is the largest correlation coefficient. Therefore, $\hat{Y}_{11}=Y_{10}+D_{8}=1.894$.

| **Day** | **Value** | **Difference** | **Cohort 1** | **Cohort 2** | **Cohort 3** | **Cohort 4** | **Cohort 5** | **CC** |
| --- | --- | --- | --- | --- | --- | --- | --- | --- |
| **1** | 1.871 |  | 1.871 |  |  |  |  |  |
| **2** | 2.073 | 0.202 | 2.073 | 2.073 |  |  |  |  |
| **3** | 1.917 | -0.156 | 1.917 | 1.917 | 1.917 |  |  |  |
| **4** | 1.930 | 0.013 | 1.930 | 1.930 | 1.930 | 1.930 |  | -0.510 |
| **5** | 2.013 | 0.083 |  | 2.013 | 2.013 | 2.013 | 2.013 | 0.280 |
| **6** | 1.967 | -0.046 |  |  | 1.967 | 1.967 | 1.967 | 0.543 |
| **7** | 1.930 | -0.037 |  |  |  | 1.930 | 1.930 | -0.694 |
| **8** | 2.001 | 0.071 |  |  |  |  | 2.001 | 0.149 |
| **9** | 2.059 | 0.058 |  |  |  |  |  |  |
| **10** | 1.823 | -0.236 |  |  |  |  |  |  |

# Supplement 2

We focused the initial time lag analysis on Bangladesh and South Africa, which displayed successions of several, roughly evenly sized peaks. The investigation was then expanded to other countries. For a proper examination of autocorrelation (AC) and average mutual information (AMI), the length of the sliding window duration (here 100 to 400 days) as well as the time lag applied (here 10 to 90 days) require intermediate extensions. Short durations or short time lags pick up a lot of noise, whereas long durations or long time lags average out relevant fluctuations (Figure S2). We noticed that the average mutual information figures, calculated over a range of parameter values (sliding window duration, lag time), seemed to diverge before the ascent of a peak in new cases per day (Figure S3). For a given range of these parameters, we therefore assessed the minimum and maximum of the computed average mutual information values on each date. Therefrom, we calculated the daily range of AMI values and plotted the result in comparison to a suitably scaled curve of normalized new cases. For the two countries under initial evaluation, we found that the AMI range increased before the onset of a peak in new infections, particularly in the lower range of lags (Figure S4). When testing on multiple countries, the diversion of AMI values (the calculated AMI range) could frequently be observed to precede a spike in new infections, but it was not sufficiently consistent to be considered reliable (Figure S5A). Focusing on Germany (which displays a prominent AMI range peak that is not followed by an increase in new cases), we varied the values for sliding window duration and lag time. However, no improvement was achieved in using the AMI range as a predictor for new infectious cases (Figure S5B,C). Seeking more consistency, we converted the daily AMI values to their absolutes and computed the sum of these absolute AMI values for each time point over a range of sliding window durations and time lags. Indeed, these daily sums displayed peaks that predated spikes in new infections (Figure S6). While their predictive value was much improved over the AMI range, the reliability was hardly sufficient (Figure S7).

On the other hand, similar approaches to calculating and plotting the autocorrelation values over time did not reveal any predictive value (cf. Figure S3). Unlike the AMI range, which increased before the onset of a peak in new infections, the AC range did not display such property (cf. Figure S4).

**Figure S2: Univariate wavelet analysis of disease progression.** The red dashed lines indicate the normalized new cases per day. The overlayed blue lines display autocorrelation or average mutual information from short sliding windows (darkest shade) to long sliding windows (lightest shade). The columns are arranged according to the shortest lag on the left and the longest lag on the right. **A,B) Data for Bangladesh.** **C,D) Data for South Africa.** A,C) Each graph displays one lag with varying sliding window durations. B,D) Each graph displays one observation window with varying lags. A-D) the top panels display autocorrelation (ac), while the bottom panels show average mutual information (ami).

**Figure S3: Average mutual information over a range of variables.** The dashed black lines indicate the normalized new cases per day. The blue arrows point to spikes in new infections and the red arrows point to an increase in the range of AMI values over the chosen parameter range. **Top) Bangladesh.** AMI was calculated for string lengths of 100 to 350 and time lags of 15 to 90. Each time lag has its assigned color, which becomes lighter with increasing sliding window durations. **Bottom) South Africa.** AMI was calculated for string lengths of 150 to 250 and time lags of 15 to 60. Each time lag has its assigned color, which becomes lighter with increasing sliding window durations.

**Figure S4: AC and AMI range in relation to normalized new cases.** The range of AMI or the range of AC (red line) is shown together with normalized new cases (black line, scaled as indicated in the legends) in a time progression. **A,B) AMI range.** For each country, A) Bangladesh, B) South Africa, the left panels represent the AMI range over the considered sliding window durations for the time lags indicated in the legends (increasing from top to bottom). Conversely, the right panels represent the AMI range over the considered time lags for the specific sliding window duration indicated in the legend (increasing from top to bottom). In one representative graph for each country, the blue arrows point to spikes in new infections, while the red arrows point to increases in the range of AMI values over the chosen parameter range, which anticipate a surge in infected cases. **A) Bangladesh.** AMI was calculated for time lags of 15, 30,60, 90 (left) and string lengths of 100, 150, 200, 250 (right). **B) South Africa.** AMI was calculated for time lags 15, 30,60 (left) and string lengths 150, 200, 250 (right). **C,D) AC range.** For each country, C) Bangladesh, D) South Africa, the left panels represent the AC range over the considered sliding window durations for the specific time lags indicated in the legends (increasing from top to bottom). Conversely, the right panels represent the AC range over the considered time lags for the specific sliding window durations indicated in the legend (increasing from top to bottom). **C) Bangladesh.** AC was calculated for time lags of 15, 30,60, 90 (left) and string lengths of 100, 150, 200, 250 (right). **D) South Africa.** AC was calculated for time lags 15, 30,60 (left) and string lengths 150, 200, 250 (right).

**Figure S5: Trends for AMI diversion. A) Comparison of several countries.** For various string lengths and time lags, AMI was calculated, and the range for the obtained AMI values was computed day-by-day. In each graph, the blue line shows the minimum AMI value, the green line the maximum AMI value, and the red line represents the calculated range. For comparison, the black line reflects the new cases per day (7-day moving average per million inhabitants), linearly converted as indicated in the legend to fit the scale. The black arrows point to spikes in new infections. The red arrows point to increases in the range of AMI values over the chosen parameter range, which predate a spike in new cases (the black arrow to its right). The dark yellow arrows point to peaks that do not follow this pattern. **B,C) Various ranges in parameter values for Germany. B)** The AMI diversion was calculated for the indicated ranges of sliding window durations over the time lags of 15 30, 60, 90. **C)** The AMI diversion was calculated for the indicated ranges of time lags over the sliding window durations of 100 to 400 in steps of 50.

**Figure S6: AMI sums over time.** Each row depicts a country, the left column shows AMI sums for increasing lags (15-90 days), the right column displays AMI sums for increasing sliding window durations (100-400 days). The red lines represent the normalized new cases per day (scaled as indicated in the legends). Purple arrows point to their peaks. The lines dotted in shades of blue are the calculated average mutual information values. The graphs on the left show the AMI sums over a range of sliding window durations for increasing time lags (the shade becomes lighter with longer lags). Green arrows point to peaks in the AMI sums that precede (are predictive of) a spike in new infections. The graphs on the right show the AMI sums over a range of time lags for increasing sliding window durations (the shade becomes lighter with longer windows).

**Figure S7: AMI sums, examples of steep changes.** Displayed are the examples of steep changes in normalized new cases and associated changes in AMI sums for Australia, Brazil, Egypt, India, and USA. The dates for troughs and peaks were read from the source data and derived calculations. nc = normalized new cases, diff. = difference.

# Supplement 3

**Figure S8: Time series forecasting. A) Lag selection.** Lag selection based on the Bayesian information criterion (BIC) in the Australia dataset. **B-D)** From the Table, -37.10 is the smallest BIC for sample size of 60, which implies that $Y_{i-2}, Y_{i-3},Y_{i-4}$ and $Y_{i-5}$ are significant lags. Similarly, they are also significant lags when sample sizes are 120 and 200. The number of significant lags can be identified through the figures. It can be seen that the first 4 lags of each observation are significant lags when the sample sizes are 60, 120 and 200. **B)** BIC for selecting significant lags based on a sample size of 60 in the Australia dataset. **C)** BIC for selecting significant lags based on a sample size of 120 in the Australia dataset. **D)** BIC for selecting significant lags based on a sample size of 200 in the Australia dataset.

**A**

| **Sample size** | **Lags** | **BIC** |
| --- | --- | --- |
| 60 | $Y_{i-2}-Y_{i-3}$ | -31.93 |
|  | $Y_{i-2}-Y_{i-4}$ | -35.99 |
|  | $Y_{i-2}-Y_{i-5}$ | **-37.10** |
|  | $Y_{i-2}-Y_{i-6}$ | -35.52 |
|  | $Y_{i-2}-Y_{i-7}$ | -31.43 |
|  | $Y_{i-2}-Y_{i-8}$ | -30.47 |
|  | $Y_{i-2}-Y_{i-9}$ | -27.93 |
|  | $Y_{i-2}-Y_{i-10}$ | -27.51 |
|  | $Y_{i-2}-Y_{i-11}$ | -24.69 |
|  | $Y_{i-2}-Y_{i-12}$ | -21.16 |
|  | $Y_{i-2}-Y_{i-13}$ | -17.78 |
|  | $Y_{i-2}-Y_{i-14}$ | -17.60 |
|  | $Y_{i-2}-Y_{i-15}$ | -13.59 |
|  | $Y_{i-2}-Y_{i-16}$ | -10.10 |
|  | $Y_{i-2}-Y_{i-17}$ | -6.28 |
|  | $Y_{i-2}-Y_{i-18}$ | -2.35 |
|  | $Y_{i-2}-Y_{i-19}$ | 1.67 |
|  | $Y_{i-2}-Y_{i-20}$ | 5.74 |
| 120 | $Y_{i-2}-Y_{i-3}$ | -114.75 |
|  | $Y_{i-2}-Y_{i-4}$ | -130.66 |
|  | $Y_{i-2}-Y_{i-5}$ | **-131.37** |
|  | $Y_{i-2}-Y_{i-6}$ | -127.50 |
|  | $Y_{i-2}-Y_{i-7}$ | -122.75 |
|  | $Y_{i-2}-Y_{i-8}$ | -123.41 |
|  | $Y_{i-2}-Y_{i-9}$ | -121.18 |
|  | $Y_{i-2}-Y_{i-10}$ | -125.52 |
|  | $Y_{i-2}-Y_{i-11}$ | -122.35 |
|  | $Y_{i-2}-Y_{i-12}$ | -118.51 |
|  | $Y_{i-2}-Y_{i-13}$ | -114.05 |
|  | $Y_{i-2}-Y_{i-14}$ | -115.30 |
|  | $Y_{i-2}-Y_{i-15}$ | -110.52 |
|  | $Y_{i-2}-Y_{i-16}$ | -105.83 |
|  | $Y_{i-2}-Y_{i-17}$ | -101.08 |
|  | $Y_{i-2}-Y_{i-18}$ | -96.30 |
|  | $Y_{i-2}-Y_{i-19}$ | -92.56 |
|  | $Y_{i-2}-Y_{i-20}$ | -88.05 |
| 200 | $Y_{i-2}-Y_{i-3}$ | -266.89 |
|  | $Y_{i-2}-Y_{i-4}$ | -298.20 |
|  | $Y_{i-2}-Y_{i-5}$ | **-301.50** |
|  | $Y_{i-2}-Y_{i-6}$ | -299.04 |
|  | $Y_{i-2}-Y_{i-7}$ | -293.88 |
|  | $Y_{i-2}-Y_{i-8}$ | -298.23 |
|  | $Y_{i-2}-Y_{i-9}$ | -294.88 |
|  | $Y_{i-2}-Y_{i-10}$ | -300.00 |
|  | $Y_{i-2}-Y_{i-11}$ | -297.17 |
|  | $Y_{i-2}-Y_{i-12}$ | -294.79 |
|  | $Y_{i-2}-Y_{i-13}$ | -289.75 |
|  | $Y_{i-2}-Y_{i-14}$ | -290.86 |
|  | $Y_{i-2}-Y_{i-15}$ | -285.79 |
|  | $Y_{i-2}-Y_{i-16}$ | -280.55 |
|  | $Y_{i-2}-Y_{i-17}$ | -275.77 |
|  | $Y_{i-2}-Y_{i-18}$ | -270.48 |
|  | $Y_{i-2}-Y_{i-19}$ | -265.97 |
|  | $Y_{i-2}-Y_{i-20}$ | -262.67 |


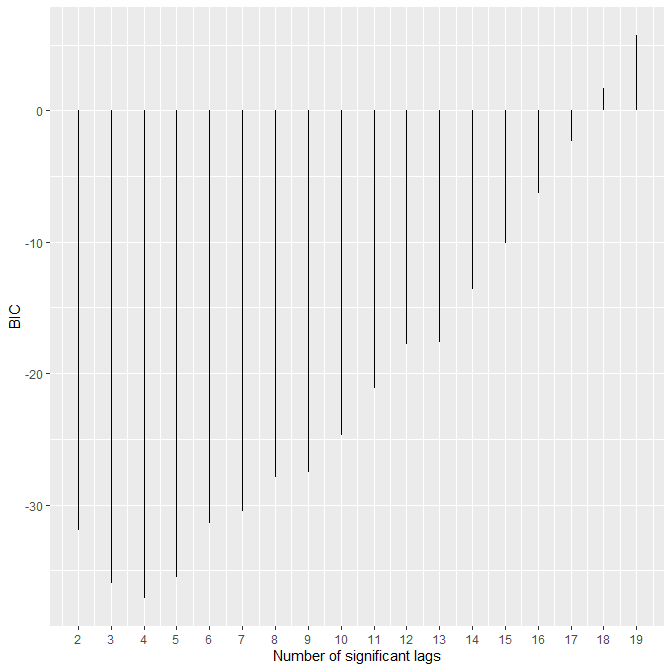


**B**

**C**

**D**


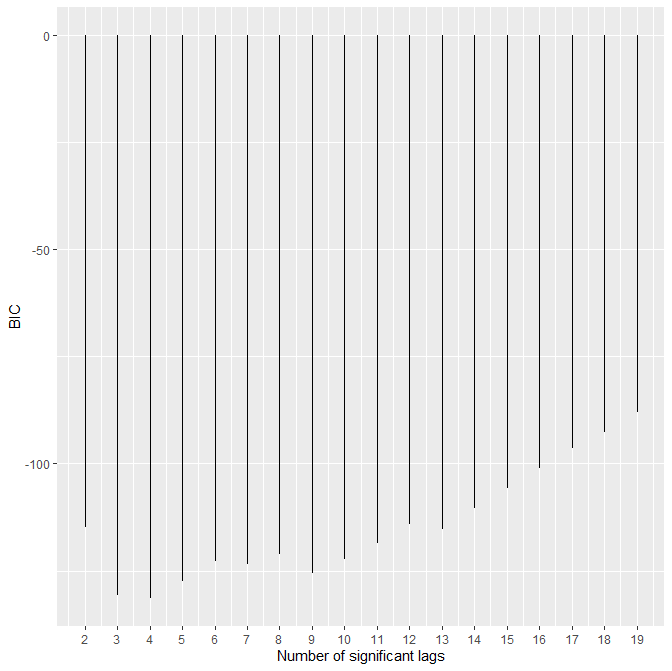


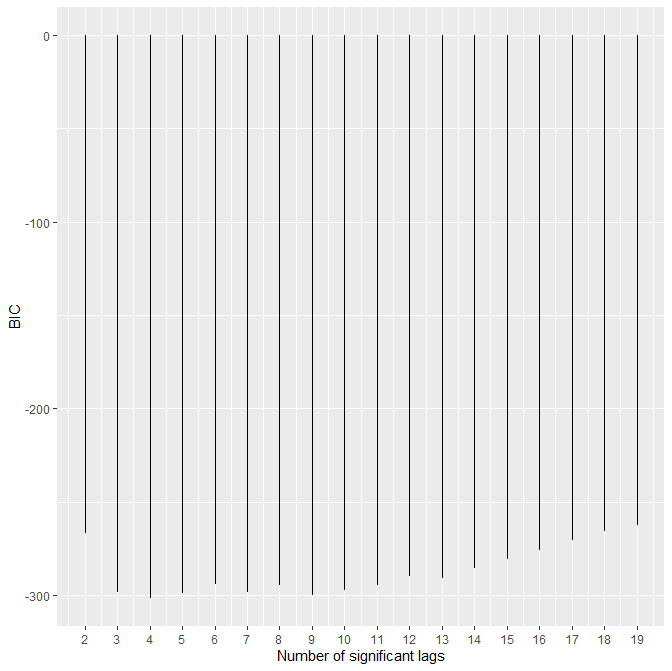


# Supplement 4

**Figure S9: Daily changes in readouts of the time series.** Approximation of a first derivative (change between consecutive days) by calculating Δ(F(x_t+1_) – F(x_t_)). **A)** For South Africa (top row), Bangladesh (middle row), Brazil (bottom row), the left panel shows the daily changes in normalized new cases and three distinct calculations of autocorrelation (all brought to comparable scales to each other as stated in the legend). The right panel displays the daily changes in normalized new cases and three distinct calculations of average mutual information (all brought to comparable scales to each other). **B)** For Bangladesh, the scaled and normalized new cases are shown (black lines), overlaid by the daily differentials in Lyapunov exponents for each axis of the 3-dimensional feature-space plot.
